# Temperature changes in the pulp chamber and bleaching gel during tooth bleaching assisted by diode laser (445 nm) using different power settings

**DOI:** 10.1007/s10103-023-03863-6

**Published:** 2023-09-12

**Authors:** Aggeliki Papadopoulou, Dimitrios Dionysopoulos, Dimitrios Strakas, Pantelis Kouros, Olga-Elpis Kolokitha, Kosmas Tolidis

**Affiliations:** 1 Department of Operative Dentistry, Faculty of Dentistry, School of Health Sciences, Aristotle University of Thessaloniki, 54124 Thessaloniki, Greece; 2 Department of Orthodontics, Faculty of Dentistry, School of Health Sciences, Aristotle University of Thessaloniki, 54124 Thessaloniki, Greece

**Keywords:** Average power, Bleaching gel, Diode laser, Intrapulpal temperature rise, Pulp health, Tooth bleaching

## Abstract

The aim of this in vitro study was to investigate the safety of using blue diode laser (445 nm) for tooth bleaching with regard to intrapulpal temperature increase operating at different average power and time settings. Fifty human mandibular incisors (*n* = 10) were used for evaluating temperature rise inside the pulp chamber and in the bleaching gel during laser-assisted tooth bleaching. The change in temperature was recorded using K thermocouples for the five experimental groups (without laser, 0.5, 1, 1.5 and 2 W) at each point of time (0, 10, 20, 30, 40, 50 and 60 s). As the average power of the diode laser increases, the temperature inside the pulp chamber also increases and that of the bleaching gel was significantly higher in all the experimental groups (*p* < 0.05). However, the intrapulpal temperature rise was below the threshold for irreversible thermal damage of the pulp (5.6 °C). Average power of a diode laser (445 nm) ranging between 0.5–2 W and irradiation time between 10–60 s should be considered safe regarding the pulp health when a red-colored bleaching gel is used. Clinical studies should confirm the safety and effectiveness of such tooth bleaching treatments. The outcomes of the present study could be a useful guide for dental clinicians, who utilize diode lasers (445 nm) for in-office tooth bleaching treatments in order to select appropriate power parameters and duration of laser irradiation without jeopardizing the safety of the pulp.

## Introduction

During the last several decades, tooth bleaching treatments have become popular in dentistry due to a greater demand on brighter teeth, which have long been considered cosmetically desirable [1]. Albeit the safety of tooth bleaching treatments has been questioned in the past [2], many studies reported that the potential adverse effects that may appear in tooth tissues are minor and reversible in a short period of time and as a result without clinical significance [3, 4]. Tooth bleaching techniques can be classified into “in-office”, which are performed at a dental office by the dentist, and “at-home”, which are implemented by the patient himself under supervision of a dentist. In-office tooth bleaching involves the use of high-concentrated bleaching gels (25–40% hydrogen peroxide or 35–38% carbamide peroxide) for shorter treatment time, and can be recommended for rapid therapy, especially in severe tooth discolorations or as a boost therapy, which might be followed by an at-home tooth bleaching technique [5].

Despite the mechanism of action of bleaching agents is still not very well clarified, it has been assumed that when the molecules of hydrogen peroxide (H_2_O_2_) diffuse into enamel are decomposed and produce free radicals, which are highly unstable because they contain one or more unpaired electrons in their atomic orbital. Thus, they tend to get an electron from the adjacent compounds to stabilize their molecular structure acting as strong oxidative agents [6]. The bleaching effect depends on the application time and composition of the bleaching gel, as well as the rate of decomposition of H_2_O_2_ molecules [7]. It has been demonstrated since 1918 that the rate of decomposition of H_2_O_2_ can be enhanced by increasing the temperature [8]. The thermal energy that is needed for decomposition of H_2_O_2_ and formation of radicals is determined by the following equation:

$${\mathrm H}_2{\mathrm O}_2+211\;\mathrm{kJ}/\mathrm{mol}\rightarrow2\mathrm{HO}$$

This can be interpreted as acceleration of H_2_O_2_ decomposition to a factor of approximately 2.2 when a temperature rise of 10 °C takes place [9]. For this reason, it has been claimed that the use of light sources during in-office tooth bleaching treatments including light emitting diodes (LEDs) and lasers might be beneficial. Dental lasers are commonly used in tooth bleaching due to clinical advantages like speed of the clinical procedure and comfort of the patient. In particular, diode lasers emitting at different wavelengths such as 810 [10], 940 [11], 960 [12] and 980 [13] nm have been investigated in previous studies for laser-assisted tooth bleaching treatments and showed clinical effectiveness regarding acceleration and improvement of tooth color change. Nevertheless, intrapulpal temperature rise has been reported during the treatments with the aforementioned laser wavelengths and ranged from 2.6 to 11.7 °C [11, 12, 14].

It is well documented that tooth pulp is a specialized connective tissue, which performs its vital functional mission within a specific temperature range. Also, it has been postulated that an intrapulpal temperature increase of 5.6ºC resulting in a critical temperature of 42.6ºC, could cause irreversible damage to pulpal tissue [15], although this threshold remains controversial in the light of most recent studies [16]. Consequently, the safety of the pulpal tissue during a laser-assisted tooth bleaching treatment is crucial for the appropriateness of the method. Recently, blue diode lasers emitting at 445 nm have also been proposed for laser-assisted tooth bleaching [17]. This laser wavelength is mainly utilized for oral surgery [18] due to its high absorption in hemoglobin and melanin [19]. Considering that pulp tissue is rich in hemoglobin, most of the light energy would be absorbed there when the laser beam is directed to the tooth. As a result, the bleaching gel should be appropriate to absorb the laser energy on the tooth surface, deterring a high intrapulpal temperature rise and improving the bleaching effect on enamel due to increase in temperature of the bleaching agent.

Therefore, the aim of this in vitro study was to investigate the safety of using a recently introduced blue diode laser (445 nm) for in-office tooth bleaching with regard to intrapulpal temperature increase operating at different average power and time settings. Additionally, the temperature of the bleaching agent applied on the tooth surface was also recorded during the treatment in order to indirectly evaluate the absorption of laser radiation from the gel. There was only one study in the literature investigated intrapulpal temperature changes during laser-assisted tooth bleaching with a diode laser emitting at 445 nm [20]. However, in that study the authors tested only two power parameters (1 and 1.5 W) at standard laser irradiation time without evaluating temperature rise in the bleaching gel.

The first null hypothesis of the study (H_0_1) stated that the increase in intrapulpal temperature during laser irradiation would not exceed 5.5 °C, which was set as the safety threshold for thermal damage of the tooth pulp. The second null hypothesis (H_0_2) stated that different power settings of the diode laser would not influence the temperature changes in the pulp chamber and in the bleaching gel.

## Materials and methods

### Sample preparation

Fifty intact human mandibular incisors, freshly extracted for periodontal reasons, were collected for this study and stored in 0.5% chloramine T solution at 6 °C for up to 3 months. Patients’ age range was around 10 years. The experiment was conducted respecting the rules of the local Ethical and Research Committee and the policies of the Aristotle University of Thessaloniki (approval No 173/25–11-2022) in accordance with the ethical standards as laid down in the 1964 Declaration of Helsinki and its later amendments. The teeth were cleaned using an ultrasonic periodontal scaler (EMS SA, CH-1260, Nyon, Switzerland) to remove the residual soft tissues and then were rinsed with slurry of pumice and water, while the labial surfaces were thoroughly polished with a prophylaxis paste (Clinpro, 3 M ESPE, St. Paul, MN, USA). Considering the variation of the natural teeth in size and shape, the sample selection was conducted in a way to ensure a maximum variation of ± 1 mm in dimensions of the teeth.

**Fig. 1:**
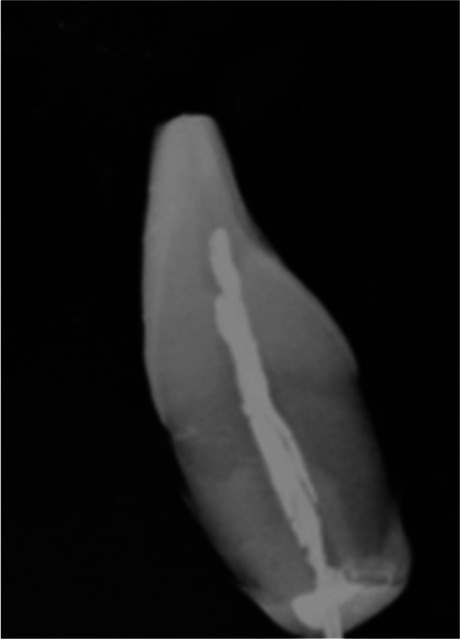
The detection of the exact location of the thermocouple inside the pulp chamber using radiographic images

The roots of the teeth were cut off approximately 4 mm from the root apex by using a high-speed water-cooled diamond disc (NTI Turbo crown cutter diamond disc C-10HP, Kerr). The root canal was enlarged mechanically up to a 2 mm diameter, using Gates-Glidden drills (sizes 1–3) till the pulp chamber. Solution of 3% sodium hypochlorite (NaOCl) followed by physiological saline was used in order to clean up the root canal and remove soft tissues and dentinal debris. The probe of the thermocouple (Type K) was pushed through the apical foramen into the coronal pulp chamber until resistance was felt, after applying a heat-transfer compound on it (Arctic silver 5, USA) Finally, the apical part was sealed with a hydrophobic flowable resin composite (Beautifil Flow Plus F03, Shofu, Kyoto, Japan), in order to ensure isolation of the root canal from water. The exact location of the thermocouple was confirmed radiographically (Fig. 1).

### Experimental set up

**Fig. 2:**
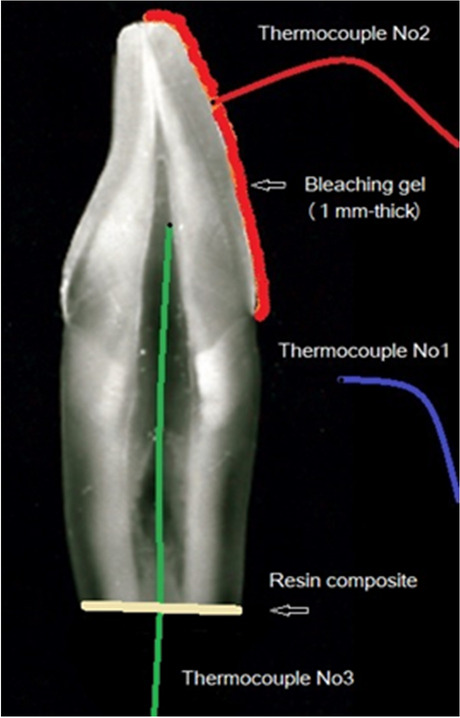
The position of the three thermocouples (No 1, 2 and 3) used in the tooth samples for recording temperature changes

Three K-type thermocouples (Dongguan Huayi Mastech Co. Ltd, Qingxi Town, Dongguan, China) were placed at the same time in each tooth sample. Thermocouple No1 (blue) measured the temperature of the room environment (23 ± 0.5 °C) and it was used as control. Thermocouple No2 (red) measured the temperature inside the bleaching gel during the procedures and thermocouple No3 (green) was measuring the temperature inside the pulp chamber (Fig. 2). The three thermocouples were connected to a thermocouple data logger (TC-08 Pico technology, Picolog 6 software, Cambridgeshire, UK) with ± 0.01 °C sensitivity and the temperature changes were recorded throughout the experimental period.

During the measurements, the tooth samples were positioned in a thermal bath (Bath WB 6 lit, Witeg Labortechnik GmbH, Wertheim, Germany) to simulate body temperature (37 ± 0.5 °C). More specifically, each tooth sample was carefully placed and stabilised on a device that allowed the whole system to be submerged into the water bath, leaving only the coronal part over the water. After the preparation of the experimental set up, a commercial in-office tooth bleaching gel (Opalescence Boost, Ultradent, South Jordan, UT, USA), with red color, containing 40% hydrogen peroxide (H_2_O_2_) was applied to the buccal tooth surface in a 1.5 mm-thick layer, which was checked with a periodontal probe.

### The experimental groups of the study

A diode laser device (SIRO Laser Blue, Sirona Dental Systems GmbH, Bensheim, Germany), which emits in continuous wave mode was tested in the current investigation. The wavelength of the laser radiation was 445 ± 5 nm, the laser tip used for the bleaching treatments was the MultiTip (lot: 0716), with a diameter of 9 mm, providing an area of 0.636 cm^2^ and generating a ‘virtual’ Gaussian TEM00 distribution. The 50 prepared tooth specimens were randomly distributed to 5 experimental groups (n = 10) according to the average output power of the laser radiation as follows:

- Group 1 (control group) – the teeth were not irradiated,
- Group 2 – the bleaching gel was irradiated up to 60 s with average output power of 0.5 W,
- Group 3: – the bleaching gel was irradiated up to 60 s with average output power of 1 W,
- Group 4: – the bleaching gel was irradiated up to 60 s with average output power of 1.5 W and
- Group 5: – the bleaching gel was irradiated up to 60 s with average output power of 2 W.

The distance between the surface of the bleaching gel and the laser tip was constantly 2.5 cm, which was achieved by the use of a custom made spacer. After laser irradiations, the thermocouples continued to record temperature change for 60 s to evaluate the rate of temperature drop. The instantaneous temperature of the thermocouples was recorded every 10 s. The power density of the laser was checked at the tip of the device using a laser power meter (LabMax-TOP; Coherent, Santa Clara, CA, USA) with a PM10 sensor (Coherent). The parameters of the laser irradiation are presented in Table 1.

**Table 1:** The parameters of the laser irradiation for each experimental group of the study

Group	Average output power	Power density	Total energy	Dose	Irradiation time
Group 1	-	-	-	-	-
Group 2	0.5 W	780 mW/cm^2^	30 J	47 J/cm^2^	60 s
Group 3	1 W	1560 mW/cm^2^	60 J	94 J/cm^2^	60 s
Group 4	1.5 W	2340 mW/cm^2^	90 J	141 J/cm^2^	60 s
Group 5	2 W	3120 mW/cm^2^	120 J	188 J/cm^2^	60 s

### Statistical analysis

Statistical analysis was performed using SPSS Statistics 27.0 (IBM Corp, Chicago, ILL, USA) software. Power analysis was used for determining the appropriate number of teeth, and the statistically minimum number of teeth was calculated as 10 for each group (*n* = 10) after conducting a pilot study. The assumption of normal distribution for the change in temperature was investigated using Shapiro–Wilk test. To compare the change in temperature (Δθ) among the five experimental groups (without laser, 0.5, 1, 1.5 and 2 W) at each point of time (0, 10, 20, 30, 40, 50 and 60 s), one-way ANOVA was used. To compare the change in temperature among the points of time (10, 20, 30, 40, 50 and 60 s) within the experimental groups, the repeated measures ANOVA test was used. Bonferroni corrections were made to adjust for multiple tests. Statistical significance level was set at *a* = *0.05*.

## Results

### Intrapulpal temperature change

**Fig. 3:**
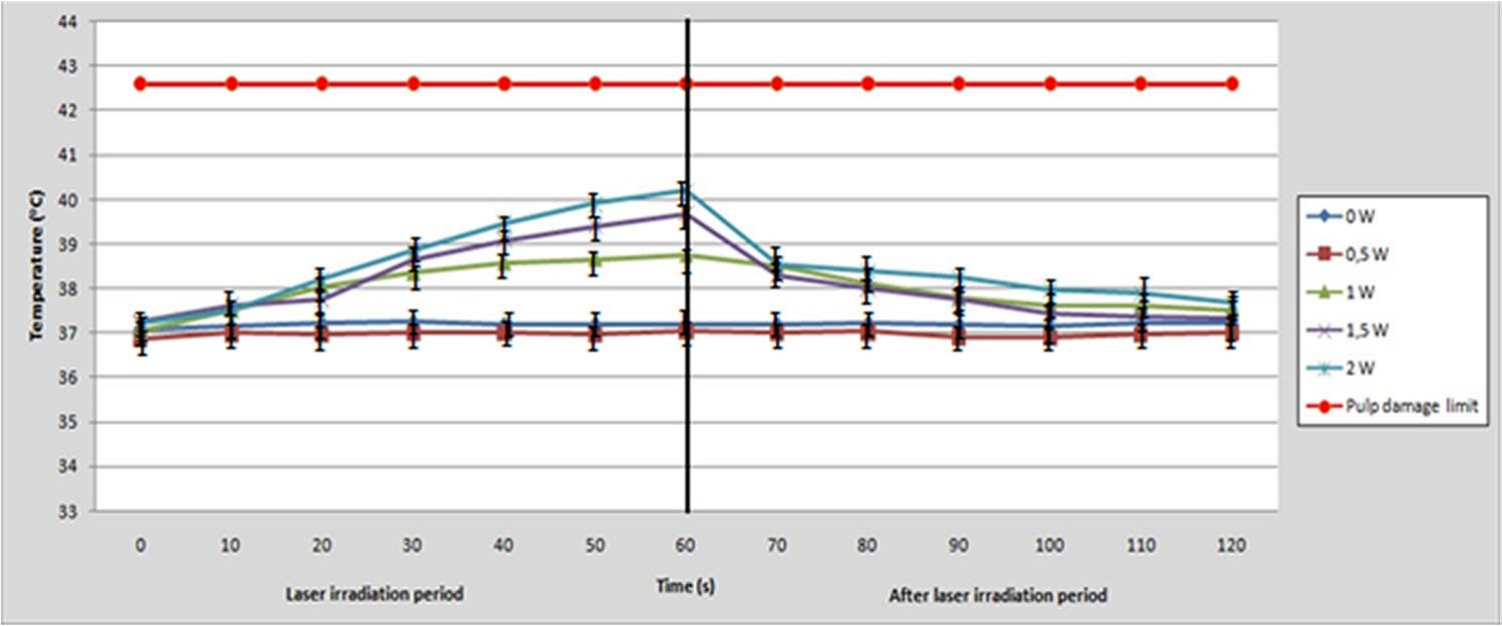
The intrapulpal temperature change during 60-s laser irradiation (left part) and 1 min after (right part) of each experimental group of the study. The red line at 42.5 °C represents the temperature limit for the safety of the tooth pulp

The change in temperature (Δθ) in degrees of Celsius (^o^C) recorded inside the pulp chamber (thermocouple No3) for each experimental group and every 10 s during laser irradiation is presented in Table 2. Also, the fluctuations in pulp chamber temperature (^o^C) during laser irradiation and 1 min after the irradiation is illustrated in Fig. 3. The results of the present study indicated that as the average power of the diode laser increases, the temperature inside the pulp chamber also increases. In particular, the statistical analysis of the data revealed significant differences among the five average power groups at each time point (*p* < 0.05) and among the six time points within each one of the five groups (*p* < 0.05). However, these differences were not clinically significant since the change in temperature did not reach the critical threshold of 5.6 °C that was adopted in the current study for irreversible histological damage of the tooth pulp [15]. In fact, during laser irradiation the highest recorded temperature was much lower than this limit (< 42.5 °C) in all the experimental groups (Fig. 3).

**Table 2:** Means and standard deviations of the intrapulpal temperature change (Δθ = θ_(t)_ – θ_(t0)_) at each point of time and for each laser power, where θ(t) = time of temperature measurement and θ(t_0_) = initial temperature at t = 0

Irradiation time	Control	0.5 W	1 W	1.5 W	2 W
10 s	0.06 (0.01)^Aa^	0.12 (0.11)^Aa^	0.48 (0.13)^Ab^	0.36 (0.19)^Ab^	0.30 (0.19)^Ab^
20 s	0.11 (0.06)^Aa^	0.11 (0.01)^Aa^	1.01 (0.20)^Bb^	0.49 (0.12)^Ac^	0.99 (0.32)^Bb^
30 s	0.15 (0.08)^Aa^	0.13 (0.03)^Aa^	1.31 (0.12) ^Cb^	1.39 (0.29)^Bb^	1.65 (0.33)^Cb^
40 s	0.10 (0.08)^Aa^	0.15 (0.06)^Aa^	1.54 (0.27) ^CDb^	1.82 (0.33)^BCbc^	2.26 (0.34)^Dc^
50 s	0.10 (0.07)^Aa^	0.10 (0.09)^Aa^	1.63 (0.33)^Db^	2.14 (0.43)^CDbc^	2.69 (0.42)^Dc^
60 s	0.08 (0.03)^Aa^	0.17 (0.14)^Aa^	1.72 (0.35)^Db^	2.42 (0.42)^Dc^	2.99 (0.44)^Dc^

Same uppercase superscripts in columns indicate no significant differences between time points measurements (*p* > 0.05)

Same lowercase superscripts in rows indicate no significant differences between average power groups (*p* > 0.05)

As it can be observed in Fig. 3 intrapulpal temperature reached a highest point at the end of laser irradiation period (after 60 s) and then gradually returned almost to its initial point after 1 min. All the laser treated groups exhibited higher intrapulpal temperature change compared to the control group (*p* < 0.05), except for 0.5 W-group which did not significantly differ (*p* > 0.05).

### Temperature change in the bleaching gel

**Fig. 4:**
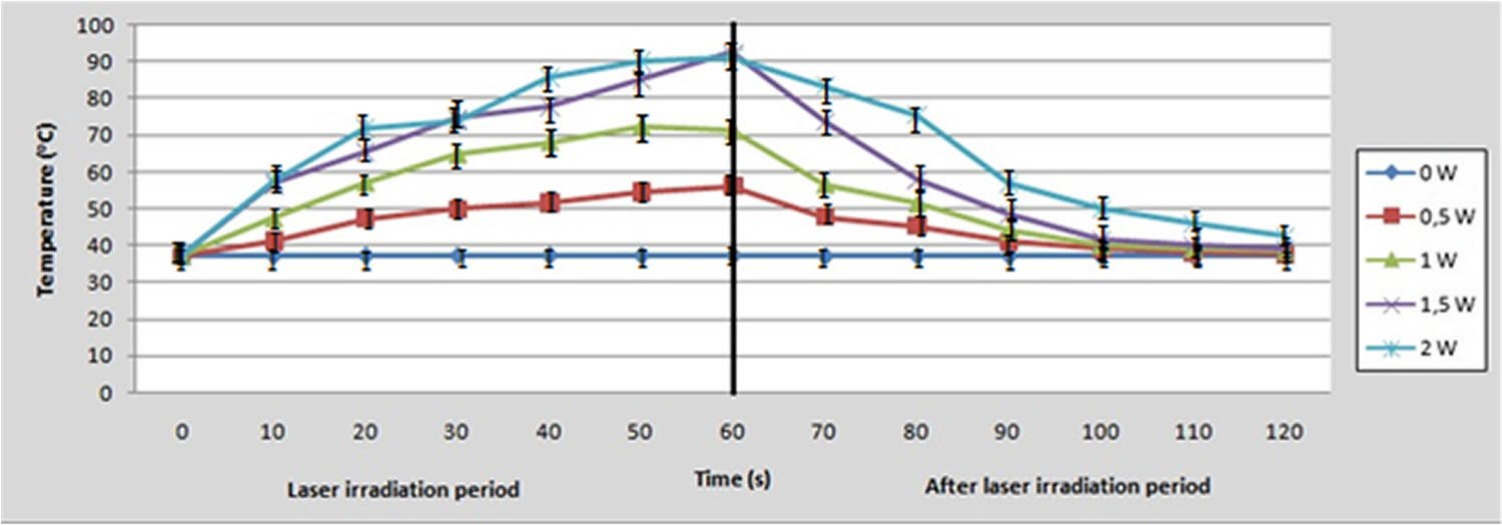
The mean temperature change of the bleaching gel during 60-s laser irradiation (left part) and 1 min after (right part) of each experimental group of the study

**Fig. 5:**
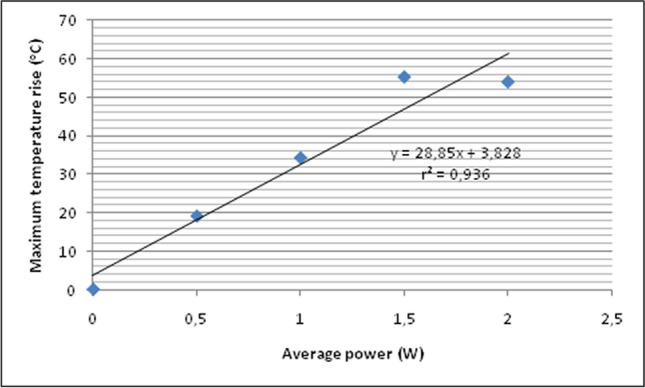
Regression analysis between average power of the laser beam and mean maximum temperature rise achieved in the bleaching gel during irradiation. A strong positive correlation can be observed as the coefficient of determination (r^2^) indicates

The change in temperature (Δθ) in ^o^C recorded in the bleaching gel (thermocouple No2) for each experimental group and every 10 s during laser irradiation is presented in Table 3. Also, the alterations in temperature (^o^C) during laser irradiation and 1 min after are illustrated in Fig. 4. There was a statistically significant increase in mean change in temperature within each group, which means that temperature increased with time during laser irradiation (*p* < 0.05). Similarly with the outcomes of the intrapulpal temperature, as the average power of the diode laser increases, the temperature in the bleaching gel increases as well. Nevertheless, this increase was significantly higher compared to the intrapulpal temperature rise, which means that most of the light energy was converted to heat in the bleaching gel as it was expected. The patterns of temperature change during laser irradiation and 1 min after were similar between the experimental groups, where the highest temperature was achieved after 60 s of laser irradiation and then a gradual decline was observed to almost the initial temperature after 1 min. Linear regression analysis revealed a strong positive correlation between average power of the laser beam and maximum temperature rise (r^2^ = 0.936), as it can be observed in Fig. 5. All the laser treated groups exhibited significantly higher temperature rise compared to the control group (*p* < 0.05).

**Table 3:** Means and standard deviations of the temperature change (Δθ = θ_(t)_ – θ_(t0)_) in the bleaching gel at each point of time and for each laser power, where θ(t) = time of temperature measurement and θ(t_0_) = initial temperature at t = 0

Irradiation time	Control	0.5 W	1 W	1.5 W	2 W
10 s	0.11 (0.03)^Aa^	4.19 (0.64)^Ab^	10.31 (1.59)^Ac^	19.58 (3.17)^Ad^	20.48 (5.29)^Ad^
20 s	0.15 (0.08)^Aa^	10.24 (1.69)^Bb^	20.14 (2.12)^Bc^	28.41 (2.95)^Bd^	34.77 (6.91)^Be^
30 s	0.11 (0.04)^Aa^	12.94 (1.56)^Bb^	27.94 (5.52)^Cc^	37.42 (5.25)^Cd^	36.65 (2.91)^Bd^
40 s	0.16 (0.09)^Aa^	14.64 (2.26)^Bb^	31.29 (6.49) ^CDc^	40.85 (5.54)^Cd^	48.66 (9.02)^Ce^
50 s	0.10 (0.02)^Aa^	17.63 (2.18) ^BCb^	35.62 (7.42)^Dc^	48.16 (7.39)^Dd^	53.03 (7.73)^Cd^
60 s	0.16 (0.07)^Aa^	19.22 (2.49)^Cb^	34.40 (5.34)^Dc^	55.46 (7.42)^Dd^	54.18 (8.47)^Cd^

Same uppercase superscripts in columns indicate no significant differences between time points measurements (*p* > 0.05)

Same lowercase superscripts in rows indicate no significant differences between average power groups (*p* > 0.05)

## Discussion

According to the results of the present investigation, H_0_1 stated that the increase in intrapulpal temperature during laser irradiation would not exceed 5.5 °C, which was set as the safety threshold for thermal damage to the tooth pulp, and this hypothesis was accepted. None of the experimental groups presented intrapulpal temperature rise over 5.5 °C, indicating that, as far as pulp health is concerned, the selected setting parameters for the diode laser (445 nm), including average power (0.5–2 W), and irradiation time (10–60 s), are clinically acceptable. This finding is in agreement with Saberi et al. [20], who also reported that the temperature increase during in-office tooth bleaching with a diode laser (445 nm) was within a safe range operating at 1 and 1.5 W average power.

It is beyond doubt that an iatrogenic increase in pulpal temperature is undesirable, as it may cause irreversible damage to pulp tissues. Several previous studies regarding the effect of temperature rise on the health of tooth pulp have adopted 5.6 °C as the thermal damage threshold, as reported by Zach and Cohen [15]. In that study, it was found that an intrapulpal temperature rise of 5.6 °C induced irreversible damage in 15% of the tested Rhesus monkeys, while at an intrapulpal temperature rise of 11 °C this percentage reached to 60%, and for temperature elevations over 16.5 °C, all the animals (100%) presented irreversible pulp tissue damage. However, the critical point for irreversible thermal damage of the pulp is still controversial.

It has been claimed that the aforementioned study is old and the method has been criticized from many researchers, who failed to obtain loss of pulp vitality at this temperature threshold and suggest a higher critical point for pulpal health [16]. Nyborg and Brännström [21] demonstrated that pathological alterations with aspiration and loss of odontoblasts may be observed in all teeth subjected to additional thermal energy. Moreover, in a more recent investigation [16] it has been found that intrapulpal temperature rise of approximately 11.2 °C did not show necrotic or reparative alterations in tooth tissues. These differences among the studies in the long-term outcome may be due to the different nature of heat application and methodology.

In another research [22], the authors focused on the detection of inflammatory mediators induced by heat as a noxious stimulus. The outcomes of this study revealed a significant increase in the synthesis of leukotriene B4 within the pulp cell cultures exposed to temperature rise up to 7 °C [23]. Notwithstanding the levels of inflammatory mediators, such as leukotriene B4, that can induce significant inflammation and irreversible tissue damage remain unknown, the clinical procedures that result in their synthesis should be avoided.

In the current investigation, a blue diode laser emitting at 445 ± 5 nm in continuous wave was tested for its appropriateness for in-office tooth bleaching treatments. The laser beam is a monochromatic coherent radiation, meaning that the wavelengths of the laser light are in phase in space and time [24]. This wavelength (445 nm) is highly adsorbed by pigmented cells like melanin (μ_A(Melanin)_ = 1033 cm^−1^) [25, 26] and hemoglobin (μ_A(Hemoglobin)_ = 1404 cm^−1^) [25, 27], while the absorption from water molecules (μ_A(Water)_ = 2.8·10^−4^ cm^−1^) [28] and hydroxyapatite (μ_A(HAp)_≈4 cm^−1^) [29] of the tooth is negligible. As a matter of fact, during irradiation of a vital tooth, most of the laser energy is absorbed by pulpal tissue, which is rich in hemoglobin leading to high temperature rise that may damage the pulp health of the tooth. For this reason, the color and composition of the bleaching gel that used during laser-assisted tooth bleaching are crucial, not only for the effectiveness of the method but primarily for the safety of the pulp [30].

As mentioned before, the tested diode laser device irradiates a blue beam (445 nm). The complementary color for blue is orange or orange-red; therefore, the selected bleaching agent in the current study included specific dyes in the orange-red band to achieve light energy absorption. Consequently, the blue laser light accelerated the bleaching process through photothermal and photochemical mechanisms, as the light energy was absorbed by the coloring agents while simultaneously preventing the penetration of laser irradiation through the tooth. Specifically, when a bleaching gel receives laser energy, a fraction of it is absorbed and the energy is converted into heat, leading to temperature rise and the consequent acceleration of the breakdown of H_2_O_2_ molecules, which increases the bleaching effect [7]. This aligns with the outcomes of the present study, which revealed a significant increase in the temperature of the bleaching gel with the lowest increase (19.22 ± 2.49 °C) at 0.5 W and the highest (55.46 ± 7.42 °C) at 1.5 W after 60 s of irradiation. Taking into consideration that a temperature rise of 10 °C accelerates decomposition of H_2_O_2_ molecules by a factor of approximately 2.2 [7], the aforementioned increases in temperature may yield an acceleration of this reaction from 4 to over 10 times. Nevertheless, in practice, the rate of decomposition of H_2_O_2_ reaches a critical point where higher temperature increase does not offer higher and faster bleaching effect [31]. Therefore, it may not be necessary to raise the temperature of the bleaching gel to a very high level, as it may not offer any advantage for the treatment, while jeopardizing the health of the tooth and surrounding tissues.

The high temperature rise of the bleaching gel recorded in the current study implies a high absorption of the laser irradiation by the specific dyes contained in the gel. Undeniably, part of the heat that is induced into the bleaching gel is transferred to the pulp chamber of the tooth through enamel and dentin. However, the temperature increase in the pulp chamber was very low and, in particular, much lower than the critical point of 5.5 °C, with the lowest increase (0.17 ± 0.14 °C) recorded at 0.5 W and the highest (2.99 ± 0.44 °C) at 2 W after 60 s of irradiation. Considering that, in clinical conditions, vital teeth contain dentinal fluid that provides more thermal insulation to the pulp, the tested laser-assisted tooth bleaching treatment could be characterized as safe for the health of the pulp under the tested experimental conditions.

Based on the outcomes of the current research, H_0_2’s statement that different power settings of the diode laser would not influence the temperature changes in the pulp chamber and in the bleaching gel was rejected. The results are consistent with previous reports, which also found discrepancies in temperature rise with different laser power settings [20, 30]. The parameters of a laser beam that determine its interaction with matter are the wavelength emitted by the laser device, the average power, and other temporal characteristics of the radiation, such as continuous versus pulsed delivery, pulse repetition, pulse duration, and time of irradiation [32]. In the present investigation, the only variable was the average power, which ranged from 0.5 to 2 W. Output power of laser radiation expresses the amount of energy induced per unit time at the tip of the handpiece of the laser device [33]. Besides, the average power of a laser beam is the average amount of work done or energy converted per unit of time [34]. From the above, it could be deduced that the higher the average power, the larger the amount of heat induced at the bleaching gel due to higher laser energy interaction with the aqueous part of the bleaching agent. This aligns with the results of the present study, which showed higher temperature rise as the average power increased from 0.5 W to 2 W. This positive correlation between average power and temperature rise was defined as strong in the current study, as indicated by the coefficient of determination (r^2^ = 0.936). Nevertheless, between 1.5 and 2 W, the temperature rise was not significantly different, and as a result, it seems reasonable to choose the lower average power (1.5 W) probably without any change in the bleaching effect.

It is important to mention that during laser irradiation, especially at higher power settings, the temperature increases to very high levels (over 90 °C), which may partially lead to evaporation of the aqueous bleaching gel. This was the reason that in the current experiment average powers over 2 W were not investigated. However, as can be noticed in the diagrams of temperature excursion, this increase lasts only a few seconds and then rapidly declines to lower levels, gradually approaching the initial temperature after 1 min. Bearing in mind that the crucial factor for thermal damage of tissues is the duration of the maintenance of the hazardous temperature, it can be concluded that due to the short period of thermal stimulus, the tested treatments are considered safe for the pulp. This is in agreement with a previous study, which reported that since the duration of temperature above the critical point for the safety of the pulp (5.5 °C) does not exceed 60 s, no irreversible damage can be occurred to the pulp tissue [35].

Strakas et al. [30], who focused on the behaviour of different teeth in temperature rise in the pulp, found that the maxillary laterals and mandibular incisors were more susceptible, presumably due to their smaller size and, as a result, the shorter distance from the tooth surface to the pulp chamber. For this reason, in the current study, the tested teeth were mandibular incisors in order to assess the worst-case scenario. Possibly, the fluctuations and differences between the teeth in temperature changes could be attributed to the variety in composition and amount of tissues among the teeth, which may lead to different heat capacity and specific heat, showing discrepancies in temperature changes.

The outcomes of the present study could be a useful guide for dental clinicians who utilize diode lasers (445 nm) for in-office tooth bleaching treatments, in order to select appropriate power parameters and duration of laser irradiation without jeopardizing the safety of the pulp. However, it should be considered that in oral conditions, these results may vary, and clinical studies are necessary to confirm their accuracy.

## Conclusions

Within the limitation of this in vitro study, it can be concluded that during tooth bleaching with a diode laser (445 nm), the temperature rise in the pulp chamber did not exceed the safety threshold of 5.5 °C for the pulp tissues. Additionally, the use of average power ranging from 0.5 W to 2 W with a 9 mm laser tip diameter, as well as irradiation time between 10 to 60 s, can be considered safe regarding the health of the pulp when a red-colored bleaching gel is used. Further studies should focus on the efficiency of tooth bleaching treatments when using these laser parameters. In particular, it should be investigated whether the optimal combinations of these parameters offer benefits compared to the conventional bleaching treatments.

## Data Availability

The data of this study are available after request to the authors.
